# Development of visual working memory precision in childhood

**DOI:** 10.1111/j.1467-7687.2012.01148.x

**Published:** 2012-07

**Authors:** Stephanie Burnett Heyes, Nahid Zokaei, Irene van der Staaij, Paul M Bays, Masud Husain

**Affiliations:** 1 Department of Experimental Psychology, University of OxfordUK; 2 UCL Institute of Cognitive Neuroscience and UCL Institute of NeurologyUK; 3 Department of Psychology, Vrije UniversiteitThe Netherlands

## Abstract

Visual working memory (VWM) is the facility to hold in mind visual information for brief periods of time. Developmental studies have suggested an increase during childhood in the maximum number of complete items that can simultaneously be stored in VWM. Here, we exploit a recent theoretical and empirical innovation to investigate instead the *precision* with which items are stored in VWM, where precision is a continuous measure reflecting VWM resolution. Ninety boys aged 7 to 13 years completed one-item and three-item VWM tasks in which stimuli were coloured bars varying in orientation. On each trial, participants used a rotating dial to reproduce the probed stimulus from memory. Results show linear age-related improvement in recall precision for both one-item and three-item VWM tasks. However, even the youngest age group stored a significant amount of information about all three items on the difficult 3-item VWM task. Importantly, the development of VWM precision was not accounted for by development on a sensorimotor control task. Whereas storage of a single complete item was previously thought to be well within the capacity limitations of the current age range, these results suggest protracted development during childhood and early adolescence in the resolution with which single and multiple items are stored in VWM. Probabilistic modelling of response distribution data suggests that improvement in VWM performance is attributable to a specific decrease in variability of stored feature representations, rather than to a decrease in misbinding or random noise. As such, we highlight a novel, potentially developmentally plausible mechanism that may underlie developmental improvement in VWM performance, independent of any alterations in the maximum number of complete items which can be stored.

## Introduction

Visual working memory (VWM) or visual short-term memory,^1^ is the facility to hold in mind visual information for brief periods of time (Luck & Vogel, 1997). This ability is considered to be a fundamental cognitive process, essential for complex reasoning, decision-making and goal-directed action (Baddeley, 2003). Developmental studies have shown that performance on well-established neuropsychological tests of VWM improves linearly during childhood (Alloway, Gathercole & Pickering, 2006; Gathercole, Pickering, Ambridge & Wearing, 2004). Typically in these paradigms, participants view a static visual array (e.g. coloured shapes) or spatiotemporal sequence of visual events (e.g. block tapping) which is held in mind during a delay. These well-validated paradigms have demonstrated, within large datasets, robust age trajectories and evidence for developmental stability in the relationship of VWM to other cognitive components (Gathercole *et al*., 2004).

However, the component mechanisms that underlie the development of VWM during childhood remain to be established (Astle & Scerif, 2011). What actually improves through development of VWM? One major focus of research has been on improvements in the *capacity* of working memory, i.e. the number of items that can be held in VWM, using visual change detection paradigms (Luck & Vogel, 1997).

In such tasks, participants judge whether a test array of visual items differs (‘change’) or does not differ (‘no change’) from a comparison array presented following a brief retention period. Test and comparison arrays are either identical, or differ by a feature of one item (e.g. colour, location). Proportion correct judgments, sensitivity (*d*’) and estimates of the maximum number of items encoded (e.g. Cowan’s *K*) increase with age during childhood (Cowan, Fristoe, Elliott, Brunner & Saults, 2006a; Cowan, Morey, AuBuchon, Zwilling & Gilchrist, 2010; Cowan, Naveh-Benjamin, Kilb & Saults, 2006b; Riggs, McTaggart, Simpson & Freeman, 2006; Riggs, Simpson & Potts, 2011).

These results suggest that improvement during childhood could be due at least in part to a discrete increase in VWM capacity, that is, the maximum number of complete items that can be held in VWM (Cowan, Elliott, Saults, Morey, Mattox, Hismjatullina & Conway, 2005; Riggs *et al*., 2006). The findings are consistent with the view that the mature visual system can maintain a maximum of 3–4 complete items in working memory ‘slots’ at any one time (Luck & Vogel, 1997).

Recently, an alternative theoretical and empirical approach to VWM has been developed in studies of adult participants. This approach investigates working memory *precision*, where precision is a continuous measure reflecting the *resolution* of items held in VWM (Bays & Husain, 2008; Bays, Wu & Husain, 2011b; Fougnie, Asplund & Marois, 2010; Gorgoraptis, Catalao, Bays & Husain, 2011; Wilken & Ma, 2004; Zhang & Luck, 2008). In precision paradigms, participants typically view either a simultaneous or a sequential array of items. Following a short delay, they are prompted to reproduce a given feature of one of the items (e.g. bar orientation) using the method of adjustment (Stevens, 1958). Precision is calculated as 1/standard deviation (*SD*) of error in response.

Crucially, VWM precision shows a robust dependency on the total number of items presented (Bays & Husain, 2008; Bays, Catalao & Husain, 2009; Gorgoraptis *et al*., 2011). Thus, with increasing memory load, each feature is stored with decreasing precision, but importantly the number of items that can be stored need not be limited: With larger set sizes, each item is stored with more variance. These findings have led to the proposal that VWM might best be considered a limited *resource* that can be distributed flexibly among memoranda, but without any limit to the *number* of complete items that can be stored (Bays & Husain, 2008; Wilken & Ma, 2004).

What happens to VWM precision during childhood? In the context of data showing discrete increases in the capacity to store complete items, it is possible that mean precision per successfully encoded item simply remains constant across age. Alternatively, precision might improve, indicating changes in the resource that can be deployed to maintain individual visual items. In the current study, we tested these competing hypotheses in a cross-sectional sample of 90 children aged 7–13 years, using a variant of a computerized, sequential VWM task that has been validated in adults (Gorgoraptis *et al*., 2011). In this paradigm, coloured bars of varying orientation were presented one at a time at a central location.

Crucially, we administered both one-item and three-item versions of the task. Developmental studies using change detection paradigms estimate that during middle childhood (e.g. age 10 years) the maximum capacity of VWM is limited to between two and three complete items (Cowan *et al*., 2006a; Riggs *et al*., 2011). To our knowledge, no previous study has shown continuing development within the current age range in VWM for a single item. In the current study, an age-associated increase in precision on the one-item VWM task would therefore provide novel evidence for the development of components other than estimated maximum capacity for complete items held in VWM. Such a result would potentially highlight a distinct developmental mechanism underlying observed improvements in VWM performance with age.

We applied a previously validated probabilistic model to characterize sources of error in VWM across age in the three-item task (Bays *et al*., 2009; Zhang & Luck, 2008). This enabled us to test whether effects of age on VWM precision are due to a change in the variability of stored features, a change in the number of items which completely fail to be stored, or some other factor, for example a change in the frequency of misbinding (Cowan *et al*., 2006a).

Because there is evidence for continuing maturation of fine motor precision and sensorimotor co-ordination across the age range of our sample (Pehoski, Henderson & Tickle-Degnen, 1997), we administered a sensorimotor control task to correct for any such factors as potential confounding effects on VWM precision estimates. Finally, to investigate the construct validity of our VWM precision measure, we investigated its relationship to indices of intelligence (IQ).

## Methods

### Participants

Ninety participants were recruited from a single-sex (male) preparatory school. Boys were selected randomly from each school year by a teacher, with the caveat that the developmental disorders dyspraxia and attention-deficit/hyperactivity disorder be excluded. Parental consent was given for each participant. The study was approved by the local ethics committee.

We excluded three boys with suspected colour blindness who could not perform a colour-naming control task or commented that ‘two bars were the same colour’ in the three-item VWM task. The final sample consisted of 87 participants aged 7.9–13.6 (mean 11.26 years, *SD* 1.48; see Table 1).

**Table 1:** Participant information

School year (grade)	*N*	Age range (years)	Mean (*SD*) age	Mean (*SD*) FSIQ^e^

3	2	7.9–8.1	8.00 (.141)	
4	14	8.8–10.3	9.30 (.440)	110.72 (13.73)
5	17	9.5–10.7	10.22 (.341)	110.16 (13.89)
6	18	9.9–11.7	11.24 (.430)	111.72 (11.70)
7	18	11.5–12.8	12.17 (.341)	115.54 (10.47)
8	18	12.8–13.6	13.22 (.260)	120.24 (11.66)

* Due to small *N* we collapsed years 3 and 4.

Standardized yearly test scores (CAT-3; http://www.gl-assessment.co.uk) were provided by the school for all but one participant. We used these scores to estimate full-scale (FS) IQ, as per Wright, Strand and Wonders (2005):
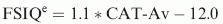
(1)where FSIQ^e^ is estimated FSIQ and CAT-Av is average CAT-3 score calculated by combining standardized scores on verbal, non-verbal and quantitative reasoning subtests (Wright *et al*., 2005).

FSIQ^e^ was correlated with age (*r*^2^ =.069, *p* =.014). Therefore, we used FSIQ^e^ as a covariate in subsequent analyses.

### Materials and measures

A colour naming task, sensorimotor control task and two visual working memory (VWM) tasks were administered on a laptop computer with the screen at a viewing distance of 60 cm. Participants were tested individually by one of two experimenters in one of two quiet rooms in school, during school hours. The duration of the experimental session was 35 minutes per participant, timed to correspond to a single 35-minute school lesson (period).

#### Colour naming task

A colour naming task was administered at the start of the experimental session. Participants were shown five screenshots from the VWM task, each containing a bar in one of five stimulus colours: red, yellow, green, blue and pink. Participants named aloud each of the five colours. Any participant who did not give 5/5 correct responses was excluded from all analyses.

#### Sensorimotor control task

**Figure 1:**
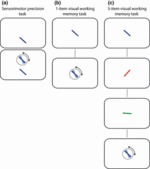
Precision tasks: (a) Sensorimotor precision control task (25 trials). A rotating dial is used to match the orientation of the probe bar (above, in circle) to that of the target bar (below). The probe bar remains on screen. (b) One-item visual working memory task (30 trials). Following a delay, the participant uses a rotating dial to match the orientation of the probe bar (below, in circle) to the remembered orientation of the target bar (above). Dotted lines denote a blank intervening delay (500 ms). (c) Three-item visual working memory task (90 trials). Following a delay, the participant uses a rotating dial to match the orientation of the probe bar (bottom, in circle) to that of the remembered orientation of the equivalently coloured target bar presented in sequence. Dotted lines denote blank intervening delays (500 ms).

Participants completed 25 trials of a sensorimotor control task (Figure 1a), directly after completion of the colour naming task. On each trial, a coloured oriented target bar (approx. 2° × 0.3° of visual angle) appeared on a grey background. After a 500 ms delay, a probe bar of the same colour appeared above the target bar, surrounded by a dark grey circle. Participants used a rotating dial (Griffin Powermate; Griffin Technology, Nashville, USA) to adjust the probe bar orientation to match that of the target, and when they were satisfied with the match they clicked the dial to proceed to the next trial. Response time was unconstrained. Note that in this task, the target bar stayed on-screen throughout the trial.

Bar colour was drawn at random on each trial from the possible colour set (red, yellow, green, blue, pink). Target and probe orientation were independently randomized across π rad on each trial. The inter-trial interval (ITI) was 500 ms.

#### Visual working memory task: one-item condition

Participants completed 30 trials of a one-item VWM task (Figure 1b), subsequent to completion of the sensorimotor control task. The task was identical to the sensorimotor control task, with the exception that a 500 ms delay (blank screen) intervened between the target and the probe stimulus, and the target and probe appeared at the same, central location. Thus, participants now rotated the probe stimulus to match the *remembered* orientation of the target.

Participants were prompted every 15 trials by the task programme to take a short break. During this break participants were encouraged to focus on a far point in the room, to minimize ocular fatigue.

#### Visual working memory task: three-item condition

Participants completed 90 trials of a three-item VWM task (see Figure 1c), subsequent to completion of the one-item task. The task had a similar format to that of the one-item VWM task. On each trial, three coloured oriented test bars appeared sequentially on the screen for a duration of 500 ms per stimulus, separated by a 500 ms blank inter-stimulus interval. Sequential rather than simultaneous presentation was used, in order to minimize the effect of potential age differences in the ability to efficiently distribute spatial attention among items in a scene (Enns & Girgus, 1985; Lane & Pearson, 1983). Bar colour was drawn at random without replacement on each trial from the possible colour set used in the colour naming task (comprising five colours). Bar orientation (stimuli, probe) was independently randomized across π rad on each trial, with the constraint that the three stimulus bars must be at a minimum of 0.175 rad separation. Following a blank 500 ms duration after the last test bar, a probe bar of the same colour as one of the test bars appeared. Participants rotated the probe bar to match the remembered orientation of the target, and the remaining stimuli were not probed. All items in the sequence were probed with equal probability.

Participants were encouraged every 15 trials to take a break. Four participants did not complete all 90 trials of the three-item task due to lack of time.

### Analysis

We investigated our dependent variables (see below) for effects of participant age and in some cases school year group (i.e. grade). Year groups 3 and 4 were collapsed due to low *N* in year group 3 (see Table 1). The pattern of results was the same when students were grouped according to age quintiles (data available on request).

Outliers > 2.5 *SD* from the sample mean of each dependent variable were excluded for that variable only (for further details, see footnotes. If it is not specified that outliers were excluded, there were no outliers). Statistical significance is *p* <.05 two-tailed unless otherwise specified.

#### Precision

For each trial of the sensorimotor, one-item VWM and three-item VWM tasks, we calculated the angular deviation between the response orientation and the original orientation of the target, i.e. the angular error. Precision was then calculated as the reciprocal of the standard deviation of error across trials (1/*SD*). Since the parameter space for orientation is circular, we used Fisher’s definition of *SD* for circular data (Fisher, 1996), subtracting the value expected for chance, so that a precision value of zero corresponds to responding at random. This method has been described previously (Bays & Husain, 2008; Bays *et al*., 2009; Bays *et al*., 2011b; Gorgoraptis *et al*., 2011), and provides a simple and intuitive measure of the fidelity with which the target orientation was reproduced.

Note that it is not justified to test for a group × task (or group × serial position) interaction in precision using repeated measures ANOVA, since precision is not expected to show additive effects across group and task (or group and serial position). Rather, it is more reasonable to assume additivity of variance, i.e. that sensorimotor and recall errors from individual and multiple items contribute independently to response variability. However, variance is not distributed normally in our tasks, so we describe below methods implemented to compare age effects across task and serial position, i.e. interactions.

At the outset we tested whether precision in the sensorimotor control task was correlated with age. A positive result entailed correction for sensorimotor precision of raw precision values in the one-item and three-item VWM tasks. Assuming that sensorimotor errors and recall errors contribute independently to response variability on VWM trials, recall precision can be estimated from the difference between VWM error variance (i.e. *SD*^2^) and sensorimotor error variance, i.e. corrected VWM precision = 1/√(*SD*_VWM_^2^–*SD*_SM_^2^). This correction is implemented throughout.

Mean one-item and three-item VWM precision values, corrected for sensorimotor performance, were interrogated for effects of participant age using linear regression and partial correlation controlling for FSIQ^e^. For the three-item VWM task, mean precision was calculated by averaging variance across serial positions of the target. We predicted effects of age on precision in the VWM tasks that were not entirely attributable to developmental improvement in sensorimotor precision. To guide interpretation of any age effects, we used one-sample *t*-tests to evaluate whether performance was above chance in each year group and task (in particular, younger year groups and the three-item VWM task).

To compare age effects on VWM precision *across tasks*, we calculated *precision differences*. This was implemented by subtracting raw one-item error variance from raw three-item error variance, and recalculating precision accordingly, i.e. precision difference = 1/√(*SD*_VWM(3)_^2^–*SD*_VWM(1)_^2^). A significant correlation between age and this dependent variable would imply a specific effect of age on storage or recall of *multiple* items in VWM, i.e. an age effect on the degree of independence of error in the recall of multiple items (e.g. age-sensitive constant attention or decay cost). An absence of association would constitute no evidence for differential rates of development between single- and multiple-item tasks.

Note that it would not be valid to interpret an interaction resulting from an ANOVA on precision across age group and task in the usual way. This is because ANOVA assumes additivity (i.e. linearity) of the dependent variable across conditions. However, precision does not behave in this way. As such, a significant interaction in repeated measures ANOVA would merely be consistent with our understanding that precision does not behave linearly; it would not necessarily indicate an interesting result. Instead, it is justified to assume additivity of *variance* (1/precision^2^), i.e. independent contribution to response variability of VWM errors from individual and multiple items. However, since response variability is not distributed normally, this violates the assumptions of ANOVA. Hence, our alternative analysis described above, the output of which is conceptually equivalent to the output of a repeated measures ANOVA on group and task.

To evaluate serial order effects, we calculated mean precision for each of the three serial positions (SP) of the three-item VWM task, i.e. first (SP1), second (SP2) and third item in sequence (SP3). Effects across the sample of the serial position of the probed item were evaluated using one-way ANOVA (within-subjects factor: SP). We expected to observe a recency effect, i.e. an advantage for the item presented last (Gorgoraptis *et al*., 2011; Hitch, Halliday, Schaafstal & Schraagen, 1988). To test for a differential effect of age on the recency effect, we calculated *precision differences* between the final (SP3) and preceding (SP1/2) items using the method outlined above. A significant result would imply age differences in the degree of independence of error in recall of multiple, serially presented items (e.g. age-sensitive constant attention or decay cost).

#### Distribution of responses

Precision gives an indication of the overall variability in responses. However, we wanted to determine how this variability was distributed across feature space. This could give some indication of sources of error in VWM. Therefore, to visualize these data we plotted the frequency of responses at each of nine arbitrary orientation bins spaced evenly across π rad of response space in the three-item VWM task, first relative to the target orientation and then relative to the non-target (unprobed) stimuli in a sequence.

However, in order to investigate more formally the distribution of responses, and thence to identify potential mechanisms underlying age-related improvement in VWM performance, we fit a probabilistic model (Bays *et al*., 2009) to each participant dataset. This model has the potential to offer insights into which aspect of VWM performance might be altering with development. Four parameters (see Figure 5a) were extracted for each participant: *κ* (kappa), a concentration parameter encapsulating Gaussian variability in memory for target orientations, and parameters representing the probability of reporting the *target* orientation, a *non-target* orientation and a random orientation. Note that reporting a non-target orientation (the orientation of a bar observed in a sequence, but different from the colour of the probed bar) would constitute a *misbinding error* (Bays *et al*., 2009). The model is described as follows:

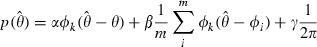
2*θ* is the true orientation of the target item, 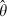 the orientation reported by the participant and φ_*k*_ is the von Mises distribution (the circular analogue of the Gaussian distribution) with mean zero and concentration parameter *κ* (kappa). The probability of reporting the target item, p(T) is given by α, the probability of reporting a non-target item and p(NT) is given by β. {ϕ_1_, ϕ_2_…ϕ_m_} are the orientations of the *m* non-target items, and the probability of responding at random, p(U) is given by γ = 1 –α - β.

Maximum likelihood estimates (Myung, 2003) of the parameters κ, α, β and γ were obtained separately for each participant for the three-item VWM task (mean across serial positions of target), using an expectation-maximization algorithm (MATLAB code available at http://www.sobell.ion.ucl.ac.uk/pbays/code/JV10/). Since the four parameters are non-independent, we tested for effects of age on each parameter separately, using linear regression and partial correlation covarying out FSIQ^e^.

#### Relationship to FSIQ^e^

We investigated the relationship between FSIQ^e^ and mean three-item VWM precision, corrected for sensorimotor precision and standardized within each year group, using linear regression.

## Results

### Sensorimotor precision improves with age

Mean precision in the sensorimotor control task (see Table 2) improved significantly with age (*r*^2^_adj_ =.179, *p* <.001). We therefore factor this out in all subsequent analyses of VWM precision. Sensorimotor precision (1/*SD* error) was consistently higher than VWM precision (see Table 2).

**Table 2:** *Mean (*SD*) precision values (rad*^−*1*^*) in each task by year group (mean age shown) and condition, excluding outliers (see text). All VWM precision values are corrected for sensorimotor precision*

	Age 9	Age 10	Age 11	Age 12	Age 13

Sensorimotor	8.08 (3.25)	8.44 (2.57)	9.00 (3.06)	12.17 (3.28)	11.93 (3.06)
One-item VWM	2.08 (1.06)	2.70 (1.37)	3.14 (1.56)	3.67 (1.70)	3.87 (1.46)
Three-item VWM	.41 (.26)	.50 (.25)	.93 (.64)	.79 (.51)	1.15 (.71)
SP1	.39 (.24)	.62 (.43)	.85 (.54)	.87 (.55)	1.10 (.81)
SP2	.52 (.33)	.60 (.49)	.98 (.63)	.76 (.46)	1.21 (.78)
SP3	1.58 (1.23)	1.40 (.95)	2.61 (1.61)	2.94 (1.62)	2.98 (1.67)

### Working memory precision improves with age on the one-item task

**Figure 2:**
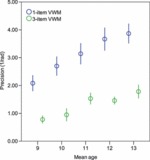
Mean precision in one-item and three-item VWM tasks, corrected for sensorimotor performance, increase linearly with age. Year group data (with mean ages) are shown for the purposes of illustration; inferential statistics were conducted using age as a continuous variable (see text).

Mean precision on the one-item VWM task improved significantly with age (*r*^2^_adj_ =.167, *p* <.001). This improvement remained significant after covarying out FSIQ^e^ (ρ =.339, *p* =.002). That is, VWM precision for a singly encoded item continues to develop between age 7 and 13 years. Precision was significantly above chance in all year groups (all *p* <.002; see Table 2, Figure 2).

### Working memory precision improves with age on the three-item task

Mean precision across the three-item VWM task,^2^ i.e. calculated by averaging variance across serial position, improved significantly with age (*r*^2^_adj_ =.156, *p* <.001). This improvement remained significant after covarying out FSIQ^e^ (ρ =.333, *p* =.002). Precision was significantly above chance in all year groups (all *p* <.001; Table 2, Figure 2) indicating that even the youngest participants encoded information in this more difficult task.

### Improvement with age is greater on the three-item VWM task

The *precision difference* between one-item and three-item VWM tasks^3^ showed an effect of age (*r*^2^_adj_ =.054, *p* =.019). This relationship remained significant after covarying out FSIQ^e^ (ρ =.187, *p* =.046 one-tailed). Thus, whereas we demonstrate significant improvement with age on both the one-item and three-item VWM tasks, the relative magnitude of improvement in VWM performance with age is greater for multiply encoded items.

In other words, the contribution to VWM variability of recall errors from multiple items becomes more independent with age, although this effect is modest. This implies a specific effect of age on storage or recall of *multiple* items in VWM. This could arise, for example, due to an age-sensitive constant attention or decay cost for each item.

### Recency and serial position effects on the three-item task

**Figure 3:**
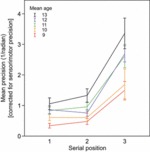
Mean precision improves with age for items presented at each serial position. Precision is highest for the most recent item in a sequence, and this effect becomes more pronounced with age. Year group data (with mean ages) are shown for the purposes of illustration; inferential statistics were conducted using age as a continuous variable (see text).

Precision improved with age for each SP of the three-item VWM task^4^ (SP1: *r*^2^_adj_ =.120, *p* =.001; SP2: *r*^2^_adj_ =.112, *p* =.001; SP3: *r*^2^_adj_ =.124, *p* =.001). This improvement remained significant after covarying out FSIQ^e^ (SP1: ρ =.324, *p* =.003; SP2: ρ =.310, *p* =.005; SP3: ρ =.327, *p* =.003). Remarkably, VWM precision at each SP was above chance in all age groups (all *p* <.001; Table 2, Figure 3). That is, even though the youngest participants show substantial immaturity in precision for single items, they are able to store some information about *each* of the three sequentially presented items.

There was an effect of target serial position (SP) on the precision of recall (*F*_1,99_ = 94.08, *p* <.001; DoF reduced to correct for non-sphericity; see Figure 3). Mean precision was higher for stimuli presented in SP3 than in SP2 (*t*_82_ = 11.54, *p* <.001) and SP1 (*t*_80_ = 11.58, *p* <.001); items presented in SP1 and SP2 did not differ significantly in precision from one another (*p* >.05). Thus, the last item in a sequence was remembered with greater precision than previous items, as expected.

The difference in precision between SP3 and SP1/2 mean showed a significant effect of age (*r*^2^_adj_ =.083, *p* =.003; covarying out FSIQ^e^: ρ =.266, *p* =.017).

### Distribution of responses

**Figure 4:**
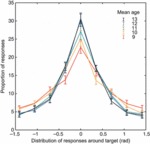
Distribution of responses with respect to target narrows with age: With increasing age, there is a decrease in variability of responses around the target orientation (three-item VWM task). This decrease in variability with age is captured within our model by the concentration parameter, kappa.

To visualize response distributions in the three-item VWM task we plotted the frequency of responses at each of nine orientation bins spaced evenly across π rad of response space with reference to target and non-target orientations. As shown in Figure 4, the variability of responses around the target alters with age (note width at half-maximum height). However, the likelihood of responding to a non-target is extremely low, and consistent across age groups (flat response distribution; not shown).

**Figure 5:**
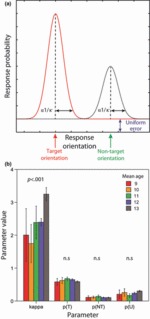
Model shows decrease in concentration parameter kappa with age: (a) Schematic showing model parameters. Kappa, the ‘concentration’ parameter corresponds to variability in feature representations: in this case orientation. p(T), p(NT) and p(U) represent the probability of responding with the recalled target, non-target or a random orientation, respectively. (b) Model parameters in each year group. The concentration parameter, kappa increases with age. No other parameter shows an effect of age. Year group data (with mean ages) are shown for the purposes of illustration; inferential statistics were conducted using age as a continuous variable (see text).

To evaluate more formally these observations, we fit a probabilistic model (Figure 5a) to each participant dataset^5^ and evaluated age effects on each of the four parameters. This showed that kappa or the ‘concentration’ parameter increased significantly with participant age (*r*^2^_adj_ =.133, *p* <.001; covarying out FSIQ^e^: ρ =.324, *p* =.003; for year group mean parameter values, see Figure 5b). Since kappa is inversely related to variance, this indicates an age-associated decrease in Gaussian VWM variance for target recall.

This was the only significant effect of age on the four model parameters. Thus there was no significant effect of age on the probability of responding to targets, responding to non-targets or making random guesses (all *p* >.05).

### Relationship between precision and IQ

**Figure 6:**
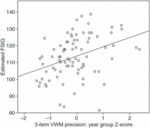
*Relationship between precision and IQ: Mean precision in the three-item visual working memory task, corrected for sensorimotor performance and standardized within year groups, is correlated with FSIQ*^*e*^.

Mean precision on the three-item VWM task, corrected for sensorimotor precision and standardized within each year group, accounted for a significant proportion of variance in FSIQ^e^ (*r*^2^_adj_ =.126, *p* =.001; Figure 6). Year-group ranked kappa was correlated with FSIQ^e^ (*r*^2^_adj_ =.023, *b* =.456, *p* =.045 one-tailed), but again this was not the case for the remaining model parameters (all *p* >.05).

## Discussion

The current study was conducted to investigate the development of *precision* in VWM during middle childhood and early adolescence. Results show that the precision with which items are recalled from VWM increases with age in a cross-sectional sample aged 7–13 years.

Importantly, this improvement in precision was observed for items presented individually (one-item VWM task), as well as in sequences of three (three-item VWM task). These effects withstood correction for the effects of age on a control task requiring fine hand–eye co-ordination, so are not readily explicable on the basis of improvement in sensorimotor factors. Thus, regardless of any putative increase in the maximum number of items that can be stored, we have shown an age-associated increase in the precision with which items – and even a single item – are stored. This is consistent with an increase in resolution of a continuous, dynamic memory resource.

Applying a probabilistic model (Bays *et al*., 2009; Gorgoraptis *et al*., 2011) to the data confirmed that the age-related increase in VWM precision was driven by a significant decrease in the variability of feature representations in working memory, rather than changes in the frequency of random guessing or misbinding errors. This finding reveals an important potential mechanism underlying developmental improvement in VWM performance, and one which in developmental terms may be more plausible than positing a step change in maximum VWM capacity.

### Development of working memory precision

Previous studies have shown improvement across childhood in performance on standard tests of VWM (Alloway *et al*., 2006; Gathercole *et al*., 2004). This improvement has been attributed to a discrete increase in capacity, that is, an increase in the maximum number of complete items that can be held in working memory (Cowan *et al*., 2005). In the current study we have shown evidence for an age-related increase in the *resolution* of items in VWM, whether presented individually or in sequences of three (Table 2; Figure 2). This represents a fundamentally different approach than estimating the maximum number of complete items encoded. Furthermore, to our knowledge, previous studies have not demonstrated development up to the age of 13 years in recall of items presented individually. Measuring precision, rather than estimating the maximum number of complete items stored, appears to be a particularly sensitive method for investigating VWM performance across age.

The effect of age on precision in the three-item VWM task amounted to a substantial decrease in SD error between the ages of 7 and 13 years (see Table 2). However, at age 13, mean performance across serial positions of the three-item VWM task (1.15 rad^−1^) remained somewhat lower than that observed previously in adults, using a similar paradigm (see Figure 3 in Gorgoraptis *et al*., 2011: specifically, solid black line, y ≍ 1.5 rad^−1^ at x = 3 items). This raises the intriguing possibility that VWM precision may continue to develop *beyond* the upper limit of the age range in the current study. The extent to which this may be due to developmental changes in attention, VWM decay or metacognition is an empirical question. Importantly, the age effects on VWM precision observed here were not accounted for by concurrent improvement on a sensorimotor control task.

Our results are consistent with findings from change detection paradigms, which show continuing development during middle childhood in estimates of the maximum number of items that may be held in VWM (Cowan *et al*., 2005; Riggs *et al*., 2006). However, our findings go beyond those from traditional measures of capacity by demonstrating protracted development throughout middle childhood and early adolescence in VWM precision for *individual* items, whether presented individually or in sequence. An implication of this finding is that childhood VWM development is best characterized not as a discrete increase in the capacity to store complete items, but by an increase in the precision with which these items are encoded, stored or retrieved.

More broadly, our findings lend weight to the notion that working memory precision offers a complementary and potentially more sensitive metric for characterizing childhood VWM performance than do traditional measures of capacity. Whilst our working memory precision findings are not directly comparable with capacity estimates (e.g. it is not possible to recover ‘total capacity’ by adding up precision per item), they complement earlier findings by providing evidence within a distinctly different empirical and conceptual framework to that of capacity, including recent reformulations of slot models (Zhang & Luck, 2008).

As a next step, potential effects of target presentation time on precision in each age group might be investigated. It is possible that younger children were detrimentally affected by the short presentation times (500 ms) of each stimulus. Whereas evidence from change detection paradigms suggests comparable childhood performance when viewing objects for 500 ms vs. 1000 ms (Cowan, AuBuchon, Gilchrist, Ricker & Saults, 2011; Cowan *et al*., 2010), recent evidence shows that continuous measures of VWM performance may be more sensitive to differences in encoding time (Bays, Gorgoraptis, Wee, Marshall & Husain, 2011a).

Of interest, our analyses showed an age-related increase in the *precision difference* between one-item and three-item VWM tasks, conceptually equivalent to an age by task interaction. Thus, whereas we demonstrate significant improvement with age on both one-item and three-item VWM tasks, consistent with age-associated development in the resolution of VWM, the relative age-associated improvement on the three-item task was more substantial. Further empirical studies are needed to identify the cognitive mechanism underlying this result. Possibilities include serial order effects (see below), emerging metacognitive capability, and the development of attention (Astle, Nobre & Scerif, 2010): for example, each additional item to be stored may incur a constant attentional cost, which is greater at younger ages.

### Serial position effects

As in previous studies investigating VWM in children and adults (Gorgoraptis *et al*., 2011; Hitch *et al*., 1988; Neath, 1993; Wright, Santiago, Sands, Kendrick & Cook, 1985), there was an effect of serial position on recall performance (Figure 3). Specifically, the final item in a sequence was recalled with higher precision than the preceding items (i.e. a recency effect), which did not differ in precision from one another. This effect became more marked with age. However, precision at *each* serial position improved significantly with age, which indicates that age effects were not solely due to an increase in precision for the most recent item.

### Modelling the distribution of responses

Previous developmental studies have shown linear improvement, during middle childhood, in standard tests of the ability to store items in VWM (Alloway *et al*., 2006; Gathercole *et al*., 2004). While our results are in agreement with these existing findings, our modelling analysis sheds new light on potential mechanisms that may underlie improvements in VWM performance.

We applied the probabilistic model described by Bays *et al*. (2009) following a related proposal by Zhang and Luck (2008), and subsequently applied within a sequential VWM task in adults by Gorgoraptis *et al*. (2011), to decompose sources of error in working memory. This analysis confirmed that the age-associated improvement in VWM performance was attributable specifically to variability in the representation of targets, and was not due to changes in the frequency of random or misbinding errors. This variability was encapsulated in our model (Figure 5a) by kappa, the ‘concentration’ parameter. The age-related increase in kappa (see Figure 5b for year group average) corresponds closely to the raw response data shown in Figure 4 (note width at half-maximum height for each year group).

Based on this finding, one important implication of our findings for brain mechanisms is that the variability of feature representations stored in VWM decreases during childhood and early adolescence. According to prominent computational neuroscience models, feature representations are implemented in the brain by population coding across neurons with tuned response properties (Pouget, Dayan & Zemel, 2000; Seung & Sompolinsky, 1993). In the case of working memory, there is evidence that this information is maintained during a delay by local recurrent excitatory networks, for example in dorsolateral prefrontal cortex (DLPFC) (Compte, Brunel, Goldman-Rakic & Wang, 2000). Progressive sharpening of these networks during development is thought to result in more *precise* feature representations (Munakata, 2004; Rolls & Deco, 2011; Schutte, Spencer & Schöner, 2003). There is evidence that such sharpening may be effected in DLPFC during childhood/adolescence by excitatory synaptic pruning (Blakemore, 2008; Gonzalez-Burgos, Kroene, Zaitsev, Povysheva, Krimer, Barrionuevo & Lewis, 2008). Such mechanisms, therefore, could potentially underlie the developmental increase in VWM precision observed in the current study.

A recent non-human primate study has linked response properties of delay neurons in DLPFC to VWM performance *decline* between young adulthood and healthy seniority (Wang, Gamo, Yang, Jin, Wang, Laubach, Mazer, Lee & Arnste, 2011). Potentially, using more sensitive precision measures in such experiments will offer important insights into developmental neurocognitive mechanisms of working memory.

### Relationship between precision and IQ

The results presented here demonstrate a positive correlation between VWM precision and full-scale IQ estimated from CAT-3 scores (FSIQ^e^; Figure 6). It has been suggested that, during development, improvements in the efficiency of complex working memory (i.e. the ability to manipulate information held in memory) underpin developmental improvements in tasks that measure the *capacity* of working memory (Fry & Hale, 2000; Gathercole & Baddeley, 1993). Therefore, it is possible that the association between FSIQ^e^ and precision observed here is underpinned by correlated improvement in higher-level processing capabilities.

### Precision and estimates of maximum capacity

Whether VWM capacity is discrete (slot models) or continuous (dynamic resource models) is debated (Bays & Husain, 2009; Cowan & Rouder, 2009). Intermediate, hybrid models propose that a dynamic resource is distributed continuously among slots (Zhang & Luck, 2008, 2009). The current data are inconsistent with a fully discrete model. Two empirical points support our interpretation of a developmental increase in precision of a dynamic resource. First, above-chance performance at *each serial position* of the three-item task suggests that even the youngest children in our sample (aged 7–9 years) succeed at encoding information about *each of the three items*. Consistent with this interpretation, there was no effect of age on the model parameter p(U), which indicates a lack of evidence that younger children were more likely than older children to respond at random on the difficult three-item task. Note that if younger children were able to store < 3 discrete items, whereas older children were able to store ≥ 3 items, for example, then this component would be expected to show an effect of age (since un-encoded items would give rise to uniformly distributed response errors).

Second, we observed robust and substantial development between age 7 and 13 years in the ability to precisely recall *each* item from a set of three, as well as development in the ability to precisely recall *an item presented individually*. This demonstrates that information is not stored in a fully discrete, all-or-none manner. Independent of any putative increase in the maximum number of complete items that can be stored, we have shown an age-associated increase in the precision with which even a single item is stored, consistent with an increase in resolution of a dynamic resource.

### Conclusions

Visual working memory is the facility to hold in mind visual objects for brief periods of time. In this study, we have shown evidence that the *precision* of VWM develops throughout middle childhood and early adolescence. This development is attributable to an increase in the resolution of feature representations held in memory, without the need to invoke alterations in the capacity to store discrete items. As such, these results demonstrate protracted development in VWM performance, and shed new light on the potential mechanisms that may underlie this development. Measuring precision, rather than estimating the maximum number of complete items that can be stored in VWM, may provide a more sensitive metric for assessing childhood VWM development. Questions remain as to whether this development reflects an increase in the ‘amount’ of VWM resource, or an increase in the extent to which VWM can be flexibly distributed across targets (Astle & Scerif, 2011).

## Footnotes

1 Some authors use the term visual short-term memory to refer to a passive store while visual working memory refers to active manipulation of items in memory. We make no such distinction here and, like some other investigators, we use visual working memory to include storage processes.

2 Three outliers > 2.5 *SD* > mean excluded.

3 Three outliers > 2.5 *SD* > mean excluded.

4 SP1: two outliers > 2.5 *SD* > mean excluded; SP2: one outlier > 2.5 *SD* > mean excluded; SP3: four outliers > 2.5 *SD* > mean excluded.

5 Subsequent to model fitting, individually outlying parameter values were excluded: three outliers > 2.5 *SD* > mean for kappa and p(U); three outliers > 2.5 *SD* < mean for p(T); four outliers > 2.5 *SD* > mean for p(NT).
